# A Bridge Too Far? An Integrative Framework Linking Classical Protist Taxonomy and Metabarcoding in Lower Termites

**DOI:** 10.3389/fmicb.2018.02620

**Published:** 2018-11-08

**Authors:** Sónia Duarte, Lina Nunes, Paulo A. V. Borges, Tania Nobre

**Affiliations:** ^1^National Laboratory for Civil Engineering, Department of Structures, Lisbon, Portugal; ^2^Evolution and Environmental Changes/Azorean Biodiversity Group, cE3c-Center for Ecology, Departamento de Ciências e Engenharia do Ambiente, Universidade dos Açores, Açores, Portugal; ^3^Laboratório de Entomologia, Instituto de Ciências Agrárias e Ambientais Mediterrânicas, Universidade de Évora, Évora, Portugal

**Keywords:** termite, protists, symbionts, metabarcoding, taxonomy

The concept of individuality has changed, since symbiosis is now accepted as being widespread and not an exception. Symbiotic microorganisms are not only crucial for the evolutionary and ecological success of many organisms (take land plants as an iconic example) but can also be key to many current human caused challenges (biomass degradation and bioenergy, for example). Nowadays, many tools are available allowing to study the hidden microbiological world, but we should not neglect that, for certain aims, researchers do need to incorporate these new technologies with less appealing and more classical approaches in an integrative framework. Using the example of lower termites' symbiosis with their protists, we intend to stimulate debate and to encourage cooperation between researchers toward high quality “big data” that can bring us closer to the pursued answers.

## The “omics era” in the microbial endosymbiosis world

The fast development of molecular techniques, especially the high-throughput sequencing methods (HTS) during the past years, has made the *in-situ* detection of microorganisms feasible. The perception that an organism is never alone is well established and fueled the desire to unveil the hidden world of the microbial symbiosis.

In this new “omics era” a new set of tools and techniques allowing the study of not yet cultivated (or difficult to cultivate) symbionts are available. The advances in DNA sequencing technology and the use of the 16S rRNA gene as a taxonomic marker have enabled the genetic identification of bacteria, being nowadays a well-established approach (e.g., Otani et al., 2014; Bin et al., 2018). For symbiotic fungi metabarcoding, approaches targeting the mycobiome of plants (e.g., mycorrhizal fungus) or invertebrates (e.g., pathogenic fungus) have been widely employed and both primer sets and comparative databases are available and growing (for a review see Cuadros-Orellana et al., 2013). For other groups, like the protists, the approaches are less well established.

In the microbial endosymbiosis world, the interactions may have several roles, from reproductive to digestive or even protective. A classic digestive endosymbiosis is the one between termites and their symbionts, and much of the research has been focused on the microbial symbiosis that aids the wood digestion process. The symbiotic association between termites and their hindgut symbionts has advantages for both, since the termites obtain energy as a result of the cooperative lignocellulose digestion, and hindgut symbionts have shelter, protection and food, supplied by the termite host (e.g., Brune, 2013; Tamschick and Radek, 2013). In addition to the synergistic digestive collaboration, symbionts of lower termites may also play a protective role against pathogens (see Peterson and Scharf, 2016).

## Symbiotic flagellate protists: the case study of lower termites

Flagellate protists inhabit the lower termite's gut, an ancestral trait shared with wood-feeding cockroaches (e.g., Lo and Eggleton, 2011; Brune and Dietrich, 2015). The gut protists belong to either the phylum Parabasalia or the order Oxymonadida (phylum Preaxostyla). Termite guts harbor a great diversity of protist species.

The characterization of flagellate protists living inside termites is a challenging wide field of research, which initially relied solely on the morphological characterization of the cells (Leidy, 1877; Kudo, 1939; Grassé, 1952), and nowadays should ideally rely on an integrative taxonomic approach, using evidence from morphology and molecular data to delimit protist species (e.g., Carpenter et al., 2009; Harper et al., 2009; James et al., 2012) and contributing to solve the Linnean shortfall (Cardoso et al., 2011; Hortal et al., 2015). However, the identification of flagellate protists symbiotic to termites is highly impaired by the difficult task of maintaining a laboratory rearing of these organisms: the intricate physical and chemical conditions existing inside the termite hindgut (particularly, gradients of oxygen, hydrogen, and pH), powered by the protists and bacterial symbionts activities, are almost impossible to reproduce in a laboratory environment, and are of utmost importance for the survival of the different species of flagellate protists. Therefore, most methods for the analysis of these symbionts are limited to the direct observation of the hindgut of freshly killed and healthy termites, followed by the isolation of each cell from the microbiome and its morphologic description. Nowadays, this is clearly not enough.

**Figure 1:**
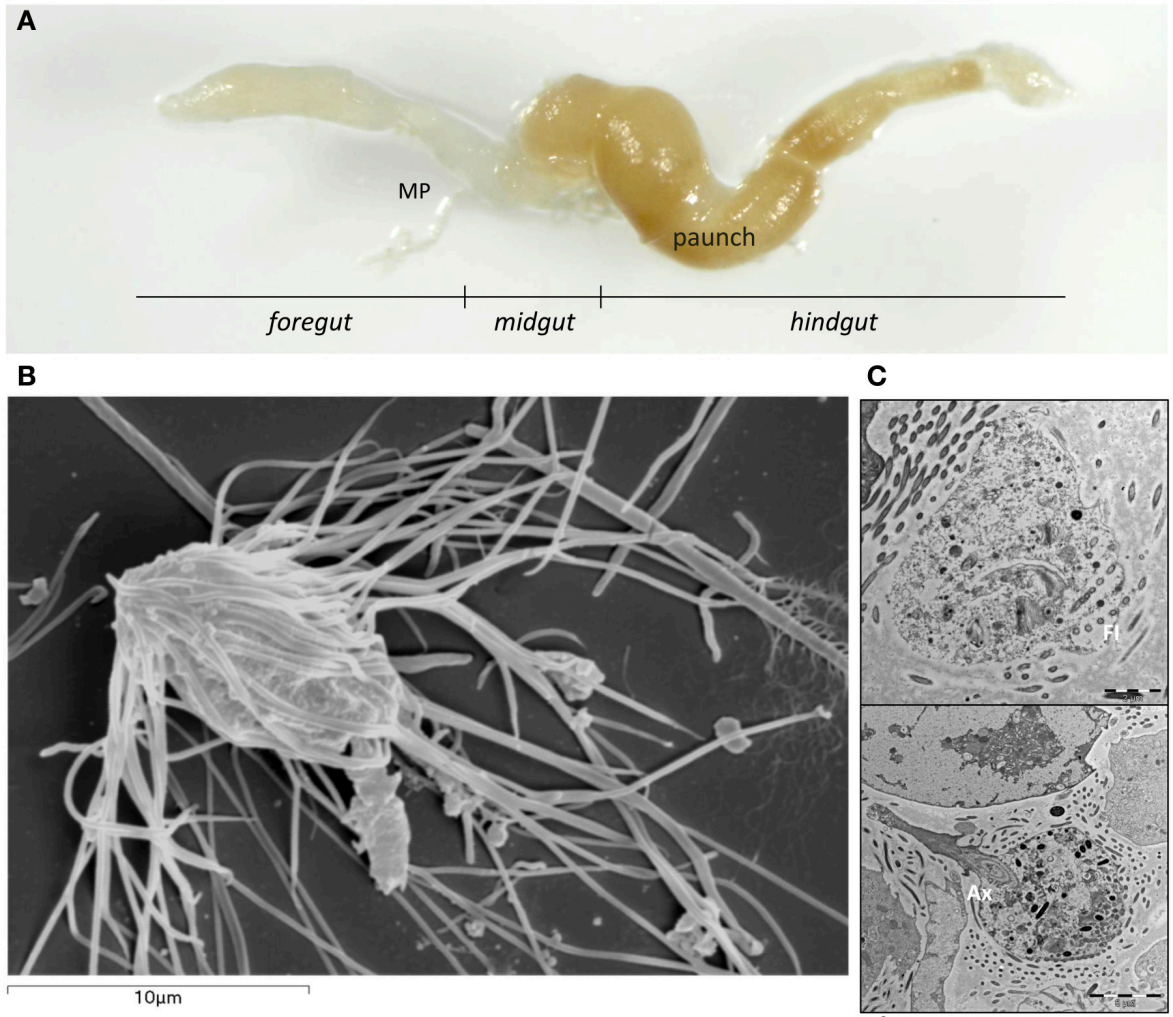
**(A)** Extracted gut with different parts of the subterranean termite *Reticulitermes grassei* Clément gut: Foregut; Midgut, including the Malpighian tubules (MP) at the posterior end of the midgut; Hindgut. **(B)** Scanning electron micrograph of *Microjoenia hexamitoides*. **(C)** Longitudinal section of *Microjoenia hexamitoides* showing flagellar lines (Fl) around the anterior zone of the cell and posteriorly protruding axostyle (Ax).

The morphologically described species, which associate to termites, amount to more than 400 parabasalids and 70 oxymonads (reviewed in Ohkuma and Brune, 2011). With the technological advances, some single cell sequences (from protists manually isolated under microscopy; Figure 1) are becoming available (Supplementary Table 1). However, many taxonomically important species have not yet been subjected to any molecular study. The *in-situ* detection of flagellate protists, through metabarcoding approaches is available but requires database data for bioinformatics comparison to obtain organisms' identification and biological role inference. Other studies, such as meta-transcriptomics are actually overcoming the drawback of the need of protists individual identification and focus on protists community of symbionts functional role by transcript inference (e.g., on lignocellulolytic process; Scharf et al., 2011; Xie et al., 2012; Raychoudhury et al., 2013, Liu et al., 2016). Even if the use of differential gene expression analyses for understanding community responses to specific conditions does overcome the need of flagellate protists species knowledge, these analyses do not contribute to our understanding of the protistan diversity nor to the identification of a given species (only community information) needed for a specific biotechnological purpose.

Furthermore, we often see no gradation in the information available, hindering the integration of the knowledge at its different scales. In short, and with some important exceptions (Supplementary Table 1), we went from single cells described morphologically to community approaches focusing on processes and responses. The relevance of this data is unarguable and could drastically increase if the “distance” between morphotypes and the operational taxonomic units or OTUs responses could be bridged.

## Bottlenecks to the workflow, from guts to flagellate protists sequences

Basically, an HTS approach to termite's protist gut community entails the sequence analysis of target amplicons, PCR amplified from DNA arising from the protist genomes directly extracted from the gut. The targets for such analyses are of course genes of taxonomic interest. The sequenced amplicons are clustered, and the representative sequences (OTUs) are compared to reference databases.

In this workflow, from guts to flagellate protists sequences, a number of bottlenecks are not yet possible to fully overcome: (a) availability of reference data (library), (b) efficient primers covering the diversity of preaxostyla and parabasalia, and (c) the lack of knowledge on the proper clustering threshold so that oxymonad and parabasalid sequences OTU do reflect a species; no doubt a consequence of the low level of knowledge on these groups of organisms.

Recently, Jasso-Selles et al. (2017) looked into the hindgut community of *Heterotermes aureus* Snyder using an integrative approach that included three techniques: (i) light microscopy, (ii) single cell isolation, and (iii) high throughput amplicon sequencing. This study is an example of bridging classical morphological studies with HTP approach, describing four new parabasalids and designed 18S rRNA gene parabasalids primers to access population-level differences in hindgut community composition. The reduced geographical range of *H. aureus* might determine its simple microbial community when compared to other Rhinotermitidae species. The characterization of the community of parabasalids symbiotic to termites belonging to *Zootermopsis* genus and the investigation of the possible coevolutionary mechanisms influencing their diversity has also been recently done following the same integrative approach (Taerum et al., 2018). *Reticulitermes* species, however, harbor flagellate protists from the phylum Parabasalia but also from the order Oxymonadida (phylum Preaxostyla) and the number of protists identified in a single *Reticulitermes* species vary per study (Lo Pinto et al., 2016 and references therein), caste and season (e.g., Benjamino and Graf, 2016) but reports of more than 15 species (or morphotypes) are accredited (Leidy, 1877; Mello, 1920; Cleveland, 1923; Bloodgood et al., 1974; Breznak and Pankratz, 1977; Mauldin, 1977; Mauldin et al., 1981; Lelis, 1992; Cook and Gold, 1998; Stingl and Brune, 2003; Stingl et al., 2005; Brugerolle, 2006; Brugerolle and Bordereau, 2006; Lewis and Forschler, 2006; Hu, 2008; Hu et al., 2011; Tamschick and Radek, 2013; and references therein: Kudo, 1939; Ghidini, 1942; Yamin, 1979; Grassé, 1982). In *Reticulitermes* at least, an HTS approach is hampered by the lack of universal primers and the absence of a reference database, including 18S rRNA gene sequences and species/morphotype linkage.

## Bridging the gap: single cell studies combining microscopy

To bridge the created gap knowledge an integrative approach should be followed, merging classical microscopy methods with single cell isolation and molecular identification. Efforts should move toward an “ID card” for every flagellate protist symbiotic of termites, where morphology, including a diagnose image and 18S rRNA gene signature should be present, together with a (tentative) taxonomy. This single locus barcoding strategy should then be followed by a more ambitious strategy to produce multi-locus protist phylogenies by sequencing several marker genes, or ideally by sequencing whole protist genomes in order to move toward performing phylogenomics of termite gut flagellates. Other information, such as morphological and/or motility characteristics, host species, geographical origin and habitat identification of the sample would be reported as extra information. If possible, molecular data on the host should be provided allowing its phylogenetic identification, and progress toward hypotheses testing, such as host-symbiont specificity and co-evolution. The tools are available; we just need to join forces! This would be the creation of a common database, at single cell level, which would enable the widespread use of meaningful and fruitful HTS approaches. Data would also be made available on all relevant databases, such as Barcode of Life Database (BOLD, for the host-http://www.boldsystems.org/; Ratnasingham and Hebert, 2007), The Protist Ribosomal Reference database (PR2-https://github.com/vaulot/pr2database; Guillou et al., 2013), SILVA ribosomal RNA database (SILVA-https://www.arb-silva.de/; Quast et al., 2013) and Eukaryotic Reference Database (EukRef-http://eukref.org/databases/; Campo et al., 2018). Describing and understanding the roles of each different species could help linking termite biology with termite control. This would also directly impact in diverse biotechnological niches, including the discovery of organisms producing lignocellulases and other enzymes -with applications in a variety of biomass, industrial, and processing technologies.

This approach will not only contribute to surpass the Linnean shortfall but will also contribute to a better understanding of the ecological and evolutionary characteristics of symbiotic microorganisms. In a near future it will be possible to document species relationships and describe the detailed networks between most symbiotic microorganisms and their hosts. More importantly this new data will populate important biodiversity databases such as the Global Biodiversity Information Facility (http://www.gbif.org/), the Catalog of Life or the Encyclopedia of Life (http://www.eol.org) and will provide invaluable resources for the advancement of ecological research and biotechnology.

## Author contributions

TN and LN conceived the idea and designed the structure of the manuscript. SD, LN, and TN drafted the manuscript, figure and supplemental material table. SD, LN, PB and TN have critically read, corrected, and approved the final version of the manuscript and agree with the opinions expressed here.

### Conflict of interest statement

The authors declare that the research was conducted in the absence of any commercial or financial relationships that could be construed as a potential conflict of interest.
