# Supplementary material for: A Bridge Too Far? An Integrative Framework Linking Classical Protist Taxonomy and Metabarcoding in Lower Termites
Source: Front Microbiol. 2018 Nov 8;9:2620. doi: 10.3389/fmicb.2018.02620 (PMC6236014; doi:10.3389/fmicb.2018.02620)
Supplement: Supplementary file 1 [file Table_1.DOCX]

A bridge too far?
An integrative framework linking classical protist taxonomy and metagenomics in lower termites

S Duarte^1,2^, L Nunes ^1^, PAV Borges^2^, T Nobre^3*^

Table 1: Summary of published works using single cell approach, whole gut approach or a combination of both (SC+WG) in the study of protists. The numbers correspond to the below references.

|  |  | **Single cell** | **Whole gut** | **SC + WG** |
| --- | --- | --- | --- | --- |
| **Microscopy** | |  |  |  |
|  | Light | 4; 8; 9; 10; | 5 | - |
|  | DIC^1^ | 2; 3; 7; 8; 11; 13; 23; 26; 28 | 21 | 12; 22; 27 |
|  | Phase-contrast | 3; 4; 6; 18 | - | - |
|  | Protargol | 26 | 21 | 22 |
|  | SEM^2^ | 4; 7; 11; 23; 25; 26 | 21 | 22 |
|  | TEM^3^ | 25; 26 | 21 | 22 |
|  | Fluorescence | 2; 26 | 5 | - |
|  | Video | 11 | - | - |
| **Gene target** | |  |  |  |
|  | SSU rRNA | 2; 3; 4; 6; 8; 9; 10; 13; 16; 18; 19; 20; 23; 25; 26; 28 | 1; 5; 21; 24; 29; 30 | 12; 15; 22; 27 |
|  | Other | actin; EF-1alfa (17); GAPDH (17; 20); ITS (27) | EF-1al4) |  |
| **Primer type** | |  |  |  |
|  | Flagellate specific | 7; 8; 13; 18; 20; 26; 28 | 1; 21; 24; 30 | 12; 15; 27 |
|  | Universal eukaryotic | 2; 3; 4; 6; 7; 8; 9; 10; 11; 16; 17; 19; 23; 25; 28 | 5; 14; 29 | 12; 22; 27 |
| **Flagellate protist group** | |  |  |  |
|  | Parabasalids | 2; 3; 4; 6; 7; 8; 9; 10; 11; 13; 16; 17; 18; 19; 20; 23; 25; 26; 28 | 1; 5; 29; 30 | 12; 22; 27 |
|  | Oxymonadids | 4; 9; 20 | 1; 21; 24 | 12; 15 |

^1^DIC - Differential interference contrast; ^2^SEM - scanning electron microscopy; ^3^TEM - Transmission electron microscopy

1. Benjamino, J., and Graf, J. (2016). Characterization of the core and caste-specific microbiota in the termite, *Reticulitermes flavipes*. *Front. Microbiol.* 7: 171. doi: 10.3389/fmicb.2016.00171
2. Boscaro, V., James, E.R., Fiorito, R., Hehenberger, E., Karnkowska, A., Del Campo, J., et al. (2017). Molecular characterization and phylogeny of four new species of the genus *Trichonympha* (Parabasalia, Trichonymphea) from lower termite hindguts. *Int. J. Syst. Evol. Microbiol.* 67(9), 3570-3575. doi: 10.1099/ijsem.0.002169
3. Del Campo, J., James, E.R., Hirakawa, Y., Fiorito, R., Kolisko, M., Irwin, N.A.T., et al. (2017). *Pseudotrichonympha leei, Pseudotrichonympha lifesoni*, and *Pseudotrichonympha pearti*, new species of parabasalian flagellates and the description of a rotating subcellular structure. *Sci. Rep.* 7: 16349. doi: 10.1038/s41598-017-16259-8
4. Desai, M.S., Strassert, J.F., Meuser, K., Hertel, H., Ikeda-Ohtsubo, W., Radek, R., et al. (2010). Strict cospeciation of devescovinid flagellates and *Bacteroidales* ectosymbionts in the gut of dry-wood termites (Kalotermitidae). *Environ. Microbiol.* 12(8): 2120-2132. doi: 10.1111/j.1462-2920.2009.02080.x
5. Gerbod D., Edgcomb V.P., Noel C., Delgado-Viscogliosi P., and Viscogliosi E. (2000). Phylogenetic position of parabasalid symbionts from the termite *Calotermes flavicollis* based on small subunit rRNA sequences. *Int. Microbiol.* 3, 165–172.
6. Gerbod, D., Nöel, C., Dolan, M.F., Edgecomb, V.P., Kitade, O., Noda, S., et al. (2002). Molecular phylogeny of parabasalids inferred from small subunit rRNA sequences, with emphasis on Devescovinidae and Calonymphidae (Trichomonadea*). Mol. Phylogenet. Evol.* 25, 545-556. doi: 10.1016/S1055-7903(02)00300-7
7. Gile, G.H., Carpenter, K.J., James, E.R., Scheffrahn, R.H., and Keeling, P.J. (2013) Morphology and molecular phylogeny of *Staurojoenina mulleri* sp. Nov. (Trichonymphida, Parabasalia) from the hindgut of the kalotermitid *Neotermes Jouteli*. *J. Eukaryot. Microbiol.* 60(2), 203-213. doi: 10.1111/jeu.12024
8. Gile, G.H, James, E.R., Tai, V., Harper, J.T., Merrell, T.L., Boscaro, V., et al. (2017) New species of *Spirotrichonympha* from Reticulitermes and the relationships among genera in Spirotrichonymphea (Parabasalia). *J. Eukaryot. Microbiol.* 65: 159-169. doi: 10.1111/jeu.12447
9. Ikeda-Ohtsubo, W., Desai, M., Stingl, U., and Brune, A. (2007). Phylogenetic diversity of 'Endomicrobia' and their specific affiliation with termite gut flagellates. *Microbiology,* 153, 3458-3465. doi: 10.1099/mic.0.2007/009217-0
10. Ikeda-Ohtsubo, W., and Brune, A. (2009). Cospeciation of termite gut flagellates and their bacterial endosymbionts: *Trichonympha* species and ‘Candidatus *Endomicrobium trichonymphae’*. *Mol. Ecol.* 18: 332-342. doi: 10.1111/j.1365-294X.2008.04029.x
11. James, E.R., Okamoto, N., Burki, F., Scheffrahn, R.H., and Keeling, P.J. (2013). *Cthulhu macrofasciculumque* n. g., n. sp. and *Cthylla microfasciculumque* n.g., n. sp., a newly identified lineage of parabasalian termite symbionts. *PLoS ONE* 8(3): e58509. doi: 10.1371/journal.pone.0058509
12. Jasso-Selles, D.E., Martini, F.D., Freeman, K.D., Garcia, M.D., Merrell, T.L., Scheffrahn, R.H., et al. (2017). The parabasalid symbiont community of *Heterotermes aureus*: Molecular and morphological characterization of four new species and reestablishment of the genus *Cononympha*. *Eur. J. Protistol.* 61, 48-63. doi: 10.1016/j.ejop.2017.09.001
13. Keeling, P.J. (2002) Molecular Phylogenetic position of *Trichomitopsis termopsidis* (Parabasalia) and evidence for the Trichomitopsiinae. *Eur. J. Protistol.* 38(3), 279-286. doi: 10.1078/0932-4739-00874
14. Moriya, S., Ohkuma, M. and Kudo, T. (1998). Phylogenetic position of symbiotic protist *Dinenympha exilis* in the hindgut of the termite *Reticulitermes speratus* inferred from the protein phylogeny of elongation factor 1α*. Gene* 210, 221-227. doi: 10.1016/S0378-1119(98)00078-X
15. Moriya, S., Dacks, J.B., Takagi, A., Noda, S., Ohkuma, M., Doolitle, W.F., et al. (2003). Molecular phylogeny of three oxymonad genera: *Pyrsonympha*, *Dinenympha* and *Oxymonas*. *J. Eukaryot. Microbiol.* 50(3), 190-197. doi: 10.1111/j.1550-7408.2003.tb00115.x
16. Noda, S., Kitade, O., Inoue, T., Kawai, M., Kanuka, M., Hiroshima, K., et al. (2007). Cospeciation in the triplex symbiosis of the termite gut protists (*Pseudotrichonympha* spp.), their hosts, and their bacterial endosymbionts. *Mol. Ecol.* 16: 1257-1266. doi: 10.1111/j.1365-294X.2006.03219.x
17. Noda, S., Mantini, C., Meloni, D., Inoue, J.I., Kitade, O., Viscogliosi, E., et al. (2012). Molecular phylogeny and evolution of Parabasalia with improved taxon sampling and new protein markers of actin and elongation factor-1α. PLoS ONE 7(1): e29938. doi: 10.1371/journal.pone.0029938
18. Noël, C., Noda, S., Mantini, C., Dolan, M.F., Delgado-Viscogliosi, P., Kudo, T., et al. (2007). Molecular phylogenetic position of the genera *Stephanonympha* and *Caduceia* (Parabasalia) inferred from nuclear small subunit rRNA gene sequences. J. Eukayot. Microbiol. 54(1): 93-99. doi: 10.1111/j.1550-7408.2006.00234.x
19. Ohkuma, M., Iida, T., Ohtoko, K., Yuzawa, H., Noda, S., Viscogliosi, E., et al. (2005). Molecular phylogeny of parabasalids inferred from small subunit rRNA sequences, with emphasis on the Hypermastigea. *Mol. Phylogenet. Evol.* 35, 646–655. doi: 10.1016/j.ympev.2005.02.013
20. Ohkuma, M., Noda, S., Hongoh, Y., Nalepa, C.A., and Inoue, T. (2009). Inheritance and diversification of symbiotic trichonymphid flagellates from a common ancestor of termites and the cockroach *Cryptocercus*. *Proc. Biol. Sci.* 276, 239–245. doi: 10.1098/rspb.2008.1094
21. Radek, R., Strassert, J.F., Krüger, J., Meuser, K., Scheffrahn, R.H., and Brune, A. (2014). Phylogeny and ultrastructure of *Oxymonas jouteli*, a rostellum-free species, and *Opisthomitus longiflagellatus* sp.nov., oxymonadid flagellates from the gut of *Neotermes jouteli. Protist* 165, 384-399. doi: 10.1016/j.protis.2014.04.003
22. Radek, R., Meuse, K., Strassert, J.F.H., Arslan, O., Teßmer, A., Šobotník, J., et al. (2017). Exclusive gut flagellates of Serritermitidae suggest a major transfaunation event in lower termites: Description of *Heliconympha glossotermitis* gen. nov. spec. nov. *J. Eukaryot. Microbiol.* 65, 77-82. doi: 10.1111/jeu.12441
23. Saldarriaga, J.F., Gile, G.H., James, E.R., Horák, A., Scheffrahn, R.H, and Keeling, P.J. (2011). Morphology and molecular phylogeny of *Pseudotrichonympha hertwigi* and *Pseudotrichonympha paulistana* (Trichonymphea, Parabasalia) from neotropical rhinotermitids. *J. Eukaryot. Microbiol.* 58(6), 487-496. doi: 10.1111/j.1550-7408.2011.00575.x
24. Stingl, U., and Brune, A. 2003. Phylogenetic diversity and whole-cell hybridization of oxymonad flagellates from the hindgut of the wood-feeding lower termite *Reticulitermes flavipes*. *Protist* 154, 147-155. doi: 10.1078/143446103764928530
25. Strassert, J.F.H., Desai, M.S., Brune, A., and Radek, R. (2009). The true diversity of devescovinid flagellates in the termite *Incisitermes marginipennis*. *Protist* 160, 522–535. doi: 10.1016/j.protis.2009.04.002
26. Strassert, J.F., Desai, M.S., Radek, R., and Brune, A. (2010). Identification and localization of the multiple bacterial symbionts of the termite gut flagellate *Joenia annectens*. *Microbiology* 156, 2068–2079. doi: 10.1099/mic.0.037267-0
27. Taerum, S.J., Martini, F.D., Liebig, J., and Gile, G.H. (2018). Incomplete co-cladogenesis between *Zootermopsis* termites and their associated protists. *Environ. Entomol.* 47(1), 184-195. doi: 10.1093/ee/nvx193
28. Tai, V., James, E.R., Perlman, S., and Keeling, P.J. (2013). Single-cell DNA barcoding using sequences from the small subunit rRNA and internal transcribed spacer region identifies new species of *Trichonympha* and *Trichomitopsis* from the hindgut of the termite *Zootermopsis angusticollis. PLoS ONE* 8(3), e58728. doi: 10.1371/journal.pone.0058728
29. Tai, V., James, E.R., Nalepa, C.A., Scheffrahn, R.H., Perlman, S.J., Keeling, P.J., et al. (2015). The role of host phylogeny varies in shaping microbial diversity in the hindguts of lower termites*. Appl. Environ. Microbiol.* 81, 1059–1070. doi: 10.1128/AEM.02945-14
30. Waidele, L., Korb, J., Voolstra, C.R., Künzel, S., Dedeine, F., and Staubach, F. (2017). Differential ecological specificity of protist and bacterial microbiomes across a set of termite species. *Front. Microbiol.* 8: 2518. doi: 10.3389/fmicb.2017.02518
